# Genome Sequence of *Moraxella macacae* 0408225, a Novel Bacterial Species Isolated from a Cynomolgus Macaque with Epistaxis

**DOI:** 10.1128/genomeA.00188-12

**Published:** 2013-02-14

**Authors:** Jason T. Ladner, Chris A. Whitehouse, Galina I. Koroleva, Gustavo F. Palacios

**Affiliations:** Center for Genomic Sciences, U.S. Army Medical Research Institute of Infectious Diseases (USAMRIID), Fort Detrick, Maryland, USA

## Abstract

*Moraxella macacae* is a recently described bacterial species that has been associated with at least two outbreaks of epistaxis in macaques. Here we present the first genome sequence of this novel species, isolated from a symptomatic cynomolgus macaque at the U.S. Army Medical Research Institute of Infectious Diseases.

## GENOME ANNOUNCEMENT

The genus *Moraxella* within the family *Moraxellaceae* contains at least 14 different species isolated from a variety of mammalian hosts, both terrestrial and aquatic. The most important human-pathogenic species is *M. catarrhalis*, a major cause of upper and lower respiratory tract infections, sinusitis, and childhood otitis media (1). *Moraxella macacae* is a recently described species causing epistaxis, or bloody nose syndrome, in both rhesus macaques from the Tulane National Primate Research Center and cynomolgus macaques from the U.S. Army Medical Research Institute of Infectious Diseases (USAMRIID) (2). Previous outbreaks of epistaxis among cynomolgus macaques have been attributed to *Branhamella* (*Moraxella*) *catarrhalis* (3) or *Neisseria* (*Moraxella*) *catarrhalis* (4) based on biochemical testing. While the results of phenotypic and biochemical testing of *M. macacae* were consistent with those of *M. catarrhalis*, sequence analysis of the 16S rRNA indicated only 88% identity to *M. catarrhalis* (2). In fact, the closest 16S sequence match was to *M. lincolnii*, a human respiratory tract inhabitant, with 90% nucleotide identity (2).

In the present study, *Moraxella macacae* 0408225, a strain isolated from a cynomolgus macaque (*Macaca fascicularis*) at USAMRIID that demonstrated sneezing with clear to serosanguinous nasal discharge, was chosen for use to sequence and annotate the genome. Genomic DNA was purified using the Qiagen DNeasy blood and tissue kit (Valencia, CA). Whole-genome sequencing was done using a combination of the Illumina GA (paired-end 76-bp reads; ~360× coverage), 454 GSFLX (6-kb mate pairs; ~80× coverage), and Pacific Biosciences (PacBio; 6-kb and 10-kb libraries, ~150× coverage each) platforms. An initial assembly was conducted using Ray (5) with the Illumina and 454 sequences, resulting in five high-quality contigs of ≥500 bp. One contig was found to be an extrachromosomal plasmid, and the entire sequence of this plasmid was verified through Sanger sequencing. The PacBio continuous-long-read [CLR] sequences were then leveraged to join the remaining four contigs using AHA (Pacific Biosciences). Finally, small assembly errors were corrected through an iterative process of mapping the Illumina reads onto the final contigs and then creating a new consensus using Bowtie2, Samtools, and custom scripts (6, 7). The final assembly consists of two chromosomal contigs (1,213,696 and 865,593 bp) and one plasmid (5,375 bp). The genome is estimated to be ~2.08 Mb, with a G+C content of 39.7%. Automated annotation by NCBI’s Prokaryotic Genomes Automatic Annotation Pipeline (PGAAP) revealed 12 rRNA genes, 46 tRNA genes, and 1,828 protein-coding sequences (CDS).

The plasmid encodes a replicase, two mobilization proteins (MobA and MobC), and one hypothetical protein but carries no known virulence factors or antibiotic resistance genes. Results from comparative genomic analysis are consistent with the conclusion that *M. macacae* is a distinct species from *M. catarrhalis*. Reciprocal BLASTp analysis (E value, <1e−10) identified 1,188 (65%) putative orthologs between *M. macacae* and *M. catarrhalis*, with an average of 39.1% amino acid divergence. Additionally, based on 16S rRNA sequences, *M. macacae* falls outside of the clade that contains *M. catarrhalis*; it appears to represent a fifth distinct clade within *Moraxellaceae* (8).

### Nucleotide sequence accession numbers.

This Whole Genome Shotgun project has been deposited at DDBJ/EMBL/GenBank under the accession number ANIN00000000. The version described in this paper is the first version, ANIN01000000.
